# The relationship between responsive caregiving and child outcomes: evidence from direct observations of mother-child dyads in Pakistan

**DOI:** 10.1186/s12889-019-6571-1

**Published:** 2019-02-28

**Authors:** Elissa Scherer, Ashley Hagaman, Esther Chung, Atif Rahman, Karen O’Donnell, Joanna Maselko

**Affiliations:** 1 University of North Carolina Chapel Hill, Chapel Hill, USA; 2 Carolina Population Center, University of North Carolina Chapel Hill, Chapel Hill, USA; 3 Department of Epidemiology, Gillings School of Public Health, University of North Carolina Chapel Hill, Chapel Hill, USA; 4 Institute of Psychology, Health and Society, University of Liverpool, Liverpool, UK; 5 Center for Child and Family Health, Duke University, Durham, NC USA

**Keywords:** Child development, Responsive caregiving, Direct observation, OMCI, LMIC, Pakistan

## Abstract

**Background:**

Responsive caregiving, or interactions in which caregivers give appropriate responses to a child’s signals, is linked to improved psychosocial, cognitive and physical outcomes in children. However, much remains unknown about how responsive caregiving affects child development across cultural and socioeconomic contexts. The purpose of this study is to examine predictors of maternal responsive caregiving and investigate how these interactions are associated with children’s development.

**Methods:**

Data for the current analyses came from a longitudinal study designed to follow mothers from the third trimester through the first three years of the child’s life. To assess responsive caregiving, the Observation of Mother-Child Interaction (OMCI) measure was used to examine maternal and child behaviors during a 5-min picture book activity at 24 months. Outcomes included child height-for-age z-score and child socioemotional development, using the Ages and Stages Questionnaire-Socioemotional (ASQ-SE) in which lower scores demonstrated better development. Using mean comparisons, the effects of baseline sociodemographic factors and maternal depression on responsive caregiving were tested. Analyses utilized hierarchical linear regressions to examine cross-sectional associations between responsive caregiving and child development outcomes at 24 months. Additional analyses controlled for the Home Observation for Measurement of the Environment (HOME), a common measure in low-income contexts of caregiving, to assess whether OMCI was uniquely predictive of child outcomes.

**Results:**

Higher maternal education attainment, lower number of children, greater socioeconomic assets, and lack of maternal depression were associated with higher levels of observed responsive caregiving behaviors. Higher total OMCI scores were associated with positive child socioemotional outcomes in adjusted models (β: -0.84, 95% CI [− 1.40, − 0.29]). The finding was statistically significant, even after controlling for HOME score (β: -0.83, 95% CI [− 1.38, − 0.27]). There was no association between OMCI scores and child linear growth.

**Conclusions:**

Responsive caregiving is linked to positive child socioemotional development in rural Pakistan. Our findings suggest that incorporating responsive caregiving into child health interventions in LMIC may have valuable impacts on child socioemotional development. The OMCI may be useful in identifying important pathways for change to responsive caregiving behaviors and may be of service for future interventions that optimize child development through responsive caregiving.

**Trial registration:**

NCT02111915 (09/18/2015); NCT02658994 (01/22/2016). Trials were prospectively registered.

## Background

Socioemotional, cognitive and physical development indicators by the age of five in low- and -middle-income countries (LMIC) have been linked to poor academic performance and poverty in adulthood, which can perpetuate poverty throughout generations [1]. Responsive caregiving has emerged in multiple settings as a key parenting domain that is linked to improved physical, cognitive and psychosocial health in children in both high and lower income countries [2, 3]. Responsive caregiving can be defined as interactions in which a caregiver provides the child with proper feedback to their behaviors and signals [4, 5]. Examples of responsive caregiving include behaviors that positively encourage focus on a task with the child, as well as positive affect and positive verbal statements. The quality of these interactions is linked to child-development knowledge [4, 6], as well as the emotional availability of the caregiver [4, 7]. Responsive caregiving is thought to be essential for forming a secure attachment relationship and has been linked to improved cognitive, health, psychosocial, disease and mortality outcomes in children [3, 8]. The positive effects of responsive caregiving behaviors have been found to extend long into childhood in high income settings. For example, more frequent displays of responsive caregiving in early childhood have been linked to lower amounts of behavioral issues at three years of age [9], higher intelligence at four and twelve years of age [10, 11], and positive academic outcomes at seven years of age [3, 12]. There is also research in high income countries that suggests responsive caregiving is linked to improved child health outcomes such as decreased hospitalizations [13]. Several findings have also identified low levels of responsive caregiving as a predictive factor of worse developmental outcomes. For example, one study found that a lack of responsive caregiving behaviors in the early months of life was linked to worse socioemotional outcomes at age three [14]. Responsive caregiving might be especially important in the context of maternal depression in that low-quality responsive caregiving may be one of the pathways through which maternal depression is predictive of non-optimal child outcomes [15]. Results from a meta-analysis of 46 studies in high-income countries indicated that maternal depression was associated with negative parent behavior (d = 0.40) and disengagement (d = 0.29) with the child [16].

Within LMIC, there is a dearth of research designed to examine responsive caregiving in detail. A key reason is that the measurement of responsive caregiving has traditionally been based on the coding of videotaped interactions between the mothers and their infants in lab environments [14, 17]. Using these procedures standardizes coding and environmental variance, but using them is a time and resource intensive endeavor that poses significant challenges to accomplish in LMIC. In addition to challenges associated with resources, lab-based recording approaches may not be culturally appropriate or possible due to human resource shortages and a lack of funding to train individuals [4]. A handful of investigators have successfully observed mothers in a naturalistic setting, and these results linked specific responsive caregiving behaviors to positive outcomes such as improved parent-child communication and better child vocabulary in relation to mother’s verbal responsive caregiving [18, 19].

The majority of research related to responsive caregiving in LMIC relies on the Home Observation for Measurement of the Environment (HOME) [12]. This tool, designed to assess home environment and stimulation quality, has been used frequently in LMIC [20–22]. Two subscales of the HOME (Responsivity and Involvement) are often used to assess responsive caregiving; however, these subscales are rarely reported separately from the total HOME score, which makes it difficult to parse out the effects of responsive caregiving from the effects of the physical home environment [4, 20].

The Observation of Mother-Child Interactions (OMCI) measure was recently created and used in rural Pakistan to observe and code structured responsive caregiving interactions without the use of video-recording [4]. The OMCI tool was developed utilizing a theoretical framework by Landry, Smith, and Swank [23] to measure the frequency of four domains of maternal responsive behaviors (contingent responding, emotional-affective support, support for infant foci of attention, and language inputs) through a structured interaction activity. In the present study, responsive caregiving was characterized using a 5-min observation of mothers and children interacting with a picture book. At the beginning of the interaction, the mother was told that the observer was interested in watching her play and talk with the child using the picture book. During the interaction, the observer simultaneously coded 11 maternal and 4 child behaviors. The interaction was intended to last five minutes. If the mother stopped early (< 4 min had passed), she was asked to continue; however, if the interaction had lasted longer than 4 min, the assessor allowed the interaction to be ended prematurely. Child behaviors were also observed to assess their responsivity to the caregiver’s behaviors [4]. Tool development occurred in a series of five steps: construction of items, field testing, expert review, data collector training, and pilot use by child development assessors based in the community [4]. During tool development, OMCI scores were found to be normally distributed, to have a high inter-observer reliability (*r* = 0.85), and to have predictive validity for child outcomes including language development (*r* = 0.62) and motor development (*r* = 0.57) [4]. Additionally, the OMCI scores were found to be significantly correlated with the Responsivity (*r* = 0.27) and Involvement (*r* = 0.33) subscales of the HOME and associated with child growth [4]. The ability to assess structured interactions without the use of video may provide additional information on responsive caregiving behaviors, since direct observation has been shown to provide less biased information on responsive caregiving behaviors than self-report measures [24]. Findings from a recent study indicated that responsive caregiving, as assessed by the OMCI, mediated the effects of responsive stimulation interventions on children’s cognitive outcomes [25]. To date, few other published studies have used the OMCI to assess its relationship with child outcomes.

This study was designed towards three goals. The first goal was to describe how maternal depression and sociodemographic variables were associated with responsive caregiving as measured by the OMCI. The second goal was to assess how responsive caregiving was linked with socioemotional and growth development indicators in the second year of life. Thirdly, since there is prior evidence that the OMCI and HOME are correlated [4], this study compared results using the OMCI and the HOME Responsivity and Involvement subscales to see if there is any new information to be gained through the use of the observational strategy.

## Methods

### Study design and participants

Data for these analyses came from the Bachpan study, a birth cohort established in the context of a perinatal depression intervention cluster-RCT in rural Pakistan [26]. The study details have been published elsewhere [26]; briefly, the Patient Health Questionnaire-9 (PHQ-9) [27] was used to screen all women who were eligible for the study, including pregnant women that were married, understood one of the languages the study was carried out in (Urdu, Punjabi, or Potohari), were planning on staying within the study area, and were not in need of any immediate medical attention. All women who scored 10 or greater on the PHQ-9 (met screening criteria for depression) were invited to participate in the study, and one in three women who scored less than 10 on the PHQ-9 (did not meet screening criteria for depression) were invited to join to create equal sample sizes of depressed and non-depressed women. Intervention or control arm random assignment was based on the cluster where each woman resided, which was controlled for in these analyses. In total, 1154 women enrolled in the study at baseline, half of whom were screened as clinically depressed in their third trimester. Interviews were conducted at pregnancy (baseline), and 3, 6, 12, and 24 months post-partum. The present study involved 881 mother-child dyads evaluated at 24 months, and complete data were available for 868 dyads.

### Measures

#### Quality of mother-child interaction

Observations of responsive caregiving were assessed at 24 months of age using an adapted version of the OMCI developed by Rasheed and Yousafzai [4]. The maternal behaviors included: positive and negative affect; positive and negative touch; positive and negative verbal statements; sensitivity; pointing and asking questions; and intrusiveness and detachment. Child behaviors included: positive and negative affect; remaining focused; and using words to communicate. Throughout the interaction, observers counted each behavior and then coded that behavior as either 0: never occurred, 1: occurred infrequently (1–2 times), 2: sometimes occurred (3–4 times), or 3: occurred frequently (5+ times). Following the procedures outlined by Rasheed and Yousafzai [4], observers were trained and reached sufficient inter-rater reliability (Kappa> 0.8) before beginning the data collection. Maternal items were summed together for a maternal score; and the same was done for the child scores, with negative items reverse coded so that higher scores reflected more positive and responsive mother-child interactions. Child and maternal scores were also combined to create a total score with a theoretical range of 0 to 45.

#### Child socioemotional and growth outcomes

The key growth outcome was height-for-age z-score (HAZ) at 24 months of age [28]. Socioemotional development was assessed using the Ages and Stages Questionnaire, Socioemotional (ASQ-SE) at 24 months of age [29, 30]. The ASQ-SE consists of 26 caregiver-reported questions about the frequency of developmentally appropriate behaviors (e.g. does your child laugh or smile when you play with him/her?) with responses ‘most of the time’ (assigned 0 points), ‘sometimes’ (assigned 5 points), and ‘rarely or never’ (assigned 10 points). Greater scores indicate greater concern about the child’s behaviors.

#### Sociodemographic variables

Based on the existing literature, we identified several other key variables and potential confounders to be included in the analyses. These included maternal age, child gender, family structure, maternal education, number of living children, and socioeconomic status (SES). Maternal age at baseline was included as a continuous variable. Child gender was taken at the three-month follow-up and was treated as a binary variable. Family structure was divided into three categories: nuclear, joint/ extended, and multiple households. Joint family structure was defined as multiple families and/or generations sharing a kitchen and monetary resources; and multiple households was defined as families that live in the same compound but with separate kitchens and financial resources. Maternal education was divided into four categories: 0 years of education, 1–5 years (primary), 6–10 years (secondary), and greater than 10 years of education. Number of living children (including the index child) was identified through a child roster, and used as a continuous variable.

Household assets were summed to generate a composite asset score as a measure of SES [31] as is common in LMIC. The asset scores were generated by adding relative weights of 22 possible assets [31]. All sociodemographic variables (as well as maternal depression) were utilized to generate mean comparisons of responsive caregiving scores as measured by the OMCI and were also subsequently included as covariates in models for the relationship between the OMCI scores and child outcomes.

#### Maternal depression

Maternal depression symptoms at the 24 month interview were assessed with the Patient Health Questionnaire- 9 (PHQ-9) [27, 32]. The PHQ-9 has been validated in Pakistan using the Structured Clinical Interview for DSM disorders (SCID) in a sample of pregnant women, and shown to have sensitivity of 94.7% and specificity of 88.9% using a score of ≥10 as the criteria for depression [32].

For these analyses, mothers who did not meet criteria for depression (those who scored less than 10 on the PHQ-9) were up-weighted to create a more representative sample; that is, 1 in 3 women who did not meet criteria for depression were asked to participate in the study from the local population. Cluster-specific weights were generated and assigned to non-depressed women. Mothers who did meet criteria for depression (those who scored greater than or equal to 10 on the PHQ-9) were assigned a default weight of 1, since all women who met criteria for depression were asked to participate.

#### The home environment

The Home Observation for Measurement of the Environment (HOME) Inventory [12] is a tool used to assess the quality of a child’s environment and available stimulation and emotional support, and has been used extensively in the Pakistani context and other LMIC [20–22]. The measure is composed of six subscales rated by observations during a home visit: Responsivity, Acceptance, Organization, Learning Materials, Involvement, and Variety [33]. Higher scores are theoretically indicative of greater home environment quality. The included HOME scores were taken by the field team at 12 months. In these analyses the total score as well as the Responsivity and Involvement subscales were used.

#### Statistical analyses

We assessed sociodemographic correlates of OMCI scores using unadjusted bivariate regressions. Next, mixed effects models were built to assess the relationship between total OMCI score, as well as the maternal and child OMCI sub-domain scores separately, and the continuous child outcomes (ASQ-SE and HAZ). These analyses started with unadjusted bivariate comparisons and then moved into modeling with robust controls (maternal age, number of living children, assets, maternal education, maternal depression, child gender, and family structure). All models account for clustering arising from the sampling strategy. Sampling weights were used to make the population more representative of the underlying population at study enrollment [26]. Finally, to explore if the OMCI was uniquely predictive of child socioemotional and growth outcomes separately from the HOME, we conducted additional analyses that included the HOME Total score and its subscales. All analyses were conducted in Stata (Version 15).

## Results

### Sample characteristics

Participants in this study included 868 mother-child dyads who enrolled at baseline and had data available at the 24-month postpartum interview (Table 1). After weighting, the mean age of the mothers was 26.55 years and the mean years of education that the mothers received was 8.14 years. Approximately 49% of the children in this study were female. The majority of the mother-child dyads lived in multigenerational households, and the average number of children per household was between 2 and 3. Sixteen percent of the mothers in this study met the screening criteria for depression at 24 months. The mean OMCI Total Score was 37.68, yielding an average score of 2.51 per item. This indicates that on average, positive behaviors occurred between 4 and 5 times, and negative behaviors occurred between 0 and 2 times.

**Table 1:** Descriptive Statistics (*n* = 868)

Characteristics	n (%)^a^ or mean (SD)^a^
Child Sex	
Male	438 (51%)
Female	430 (49%)
Mother age at baseline (years)	26.55 (4.33)
18–21	108 (13%)
22–25	270 (32%)
26–29	250 (29%)
30–33	174 (19%)
34–40	66 (7%)
Meets Criteria for Depression	
No	712 (84%)
Yes	156 (16%)
Maternal Education (years)	8.14 (4.47)
0	126 (13%)
1–5 (primary)	165 (17%)
6–10 (secondary)	386 (45%)
> 10	191 (25%)
Household Structure	
Nuclear	264 (29%)
Joint or Extended	533 (63%)
Multiple Households	71 (8%)
Number of Children in Household	
1	178 (21%)
2–3	496 (58%)
4–5	166 (18%)
> 5	28 (3%)

^a^Percents and means weighted using sampling weights

### Predictors of the quality mother-child interaction

In unadjusted bivariate regressions, greater assets, higher levels of maternal education, lower numbers of living children, and lack of maternal depression were all significantly associated with higher OMCI scores (Table 2). On average, dyads from the lowest economic quintile had an OMCI score of 36.16, while those in the highest economic quintile had an average score of 38.57 (Table 2). Similarly, mothers who received no schooling had an average OMCI score of 36.20, while mothers with greater than ten years of education had an average score of 38.96 (Table 2). Additionally, dyads from families in which the infant assessed was the sole child in the family scored 38.59 on average, while dyads from families that had five or more children scored 35.51, on average (Table 2). Finally, dyads in which the mother met criteria for depression scored, on average, 37.07 on the OMCI measure, while those who did not meet criteria for depression had an average score of 37.95 (Table 2).

**Table 2:** Means and unadjusted bivariate regression comparisons for OMCI at 24 months by Baseline Sociodemographic Characteristics and Current Depression (OMCI *n* = 868)

Characteristics	Mean total OMCI Score	*P*-value
Child Gender		
Male	37.56	
Female	38.07	0.07
SES Quintile		
1 (lowest)	36.16	
2	37.84	0.00*
3	37.89	0.00*
4	38.18	0.00*
5 (highest)	38.57	0.00*
Family Structure		
Nuclear	37.50	
Joint/Extended	37.98	0.16
Multiple	37.65	0.79
Number of Living Children		
1	38.59	
2–3	37.94	0.05
4–5	36.83	0.00*
> 5	35.51	0.01*
Maternal Education		
0	36.20	
1–5 (primary)	37.43	0.03*
6-10 (secondary)	37.79	0.00*
> 10	38.96	0.00*
Maternal Age		
18–21	37.70	
22–25	37.63	0.88
26–29	38.25	0.24
30–33	37.75	0.94
34–40	37.25	0.53
Maternal Depression at 24 months		
No	37.95	
Yes	37.07	0.03*

Sampling weights and clustering were applied in all comparisons

*p < 0.05, relative to the reference

### Quality of mother-child interaction and child socioemotional and growth outcomes

The OMCI Total score, as well as the maternal and child subdomains, were significantly related to children’s socioemotional outcomes above and beyond other sociodemographic factors (Table 3). As a reminder, lower ASQ-SE scores are indicative of better child socioemotional outcomes. In fully adjusted models, for every one-point increase in the OMCI total score, there was nearly a one-point improvement (β: -0.84, 95% CI [− 1.40, − 0.29]) in the child’s ASQ-SE score. Additionally, for every one-point increase in the maternal score, there was a − 0.68 point (95% CI [− 1.30, − 0.06]) improvement in the child’s ASQ-SE score and for every one-point increase in the child score, there was a − 1.75 point (95% CI [− 2.79, − 0.70]) improvement in the child’s ASQ-SE score (Table 3).

**Table 3:** OMCI OLS regression modeling at 24 months of ASQ-SE scores at 24 months (OMCI *n* = 868)

	Unadjusted bivariate comparisons			Adjusted Model		
Separate Regressions	Beta Coef.	[95% Conf. Interval]		Beta Coef.	[95% Conf. Interval]	
1. OMCI Total Score	-0.83	−1.36	− 0.31	− 0.84	− 1.40	− 0.29
2. OMCI Maternal Score	− 0.69	− 1.27	− 0.12	− 0.68	− 1.30	− 0.06
3. OMCI Child Score	−1.74	− 2.78	− 0.70	− 1.75	− 2.79	− 0.70

Sampling weights and clustering were applied in all models

Adjusted model: Covariates include Maternal Age, Number of living children, Asset Score, Family Structure, Maternal Depression, Maternal Education, Child Gender

In unadjusted models, the OMCI Total Score was weakly associated with child height-for-age z-scores (β:0.01, 95% CI [− 0.01, 0.04]) (Table 4). In the adjusted models, all estimates were attenuated resulting in no significant associations between OMCI and child height-for-age z-scores.

**Table 4:** OMCI OLS regression modeling at 24-months on height-for-age z-score at 24-months (OMCI *n* = 868)

	Unadjusted bivariate comparisons			Adjusted model		
Separate Regressions	Beta Coef.	[95% Conf. Interval]		Beta Coef.	[95% Conf. Interval]	
1. OMCI Total Score	0.01	−0.01	0.04	0.00	−0.02	0.02
2. OMCI Maternal Score	0.02	−0.01	0.04	0.00	−0.03	0.03
3. OMCI Child Score	0.02	−0.02	0.06	0.00	−0.04	0.04

Sampling weights and clustering were applied in all models

Adjusted model: Covariates include Maternal Age, Number of living children, Asset Score, Family Structure, Maternal Depression, Maternal Education, Child Gender

Next, we modelled the association between the HOME score and ASQ-SE alone as well as in a model together with the OMCI score (Table 5). In its own model, the HOME total score but not the Responsivity or the Involvement subdomains were associated with ASQ-SE. Including any of the HOME scores (total, Responsivity, or Involvement) did not meaningfully alter the strength of the association between the OMCI and ASQ-SE. For example, controlling for the HOME Responsivity score, the estimate for OMCI maternal score was − 0.75 (95% CI [− 1.39, − 0.10]) (Table 5), while in a model without the HOME Responsivity score, the estimate was − 0.68, (95% CI [− 1.30, − 0.06]) (Table 3).

**Table 5:** HOME OLS regression modeling at 12 months on ASQ-SE scores at 24 months (HOME *n* = 840)

Adjusted HOME Models		Adjusted HOME Models with OMCI	
Separate Models	Estimate (95% CI)	Combined Models	Estimate (95% CI)
1. HOME Total Score	−0.66 (−1.05, − 0.27)	1. HOME Total Score	−0.60 (− 0.98, − 0.21)
		1. OMCI Total Score	−0.83 (− 1.38, − 0.27)
2. HOME Responsivity	−1.38 (−2.92, 0.16)	2. HOME Responsivity	− 1.33 (− 2.84, 0.19)
		2. OMCI Maternal	−0.75 (− 1.39, − 0.10)
3. HOME Involvement	0.72 (− 0.74, 2.17)		

Sampling weights and clustering were applied in all models

Adjusted models: Covariates include Maternal Age, Number of living children, Asset Score, Family Structure, Maternal Depression, Maternal Education, Child Gender

Table 6 shows the parallel analyses predicting height-for-age z-scores. Only the HOME total score was significantly associated with slightly higher height-for-age scores (β: 0.02, 95% CI [0.00, 0.04]). As in the previous models, OMCI was not associated with height-for-age z-score.

**Table 6:** HOME OLS regression modeling at 12 months on height-for-age scores at 24 months (HOME *n* = 840)

Adjusted HOME Models		Adjusted HOME Models with OMCI	
Separate Models	Estimate (95% CI)	Combined Models	Estimate (95% CI)
1. HOME Total Score	0.02 (0.00, 0.04)	1. HOME Total Score	0.02 (0.00, 0.04)
		1. OMCI Total Score	−0.01 (− 0.02, 0.02)
2. HOME Responsivity	0.04 (−0.01, 0.09)	2. HOME Responsivity	0.04 (−0.01, 0.09)
		2. OMCI Maternal	0.00 (−0.03, 0.03)
3. HOME Involvement	0.06 (−0.01, 0.12)		

Sampling weights and clustering were applied in all models

Adjusted models: Covariates include Maternal Age, Number of living children, Asset Score, Family Structure, Maternal Depression, Maternal Education, Child Gender

## Discussion

This study was designed to investigate the relationship between responsive caregiving and child outcomes using the recently developed Observation of Mother-Child Interaction (OMCI) tool. The results of this study suggest that maternal depression, maternal education, household assets, and number of children are significantly related to maternal responsive caregiving scores, pointing to the importance of sociodemographic factors in determining responsive caregiving in LMIC. Additionally, the results of this study highlight responsive caregiving as a significant factor associated with positive socioemotional outcomes for children in rural Pakistan. This finding remained statistically significant after controlling for sociodemographic factors and maternal depression, indicating that improved responsive caregiving is associated with improved child outcomes even in low-resource environments. Finally, we found that the OMCI uniquely predicts socioemotional outcomes and is distinct from the responsive caregiving HOME subscales.

Our findings regarding the sociodemographic links with responsive caregiving corroborate the current body of literature. A recent study that examined data from 44 LMIC countries found that maternal education was linked to improved child outcomes, and that a key mechanism in this association was responsive caregiving behaviors, such as positive stimulation and other behaviors that supported learning [34]. Associations have shown that improved child outcomes are linked to higher assets partially through the mediating factors of responsive caregiving behaviors [35]. Our finding between maternal depression and responsive caregiving aligns with previous bodies of literature that have connected maternal depression to negative behaviors towards the child [16] and have also linked participation in depression interventions to better physical and cognitive health outcomes for children later in life [36].

This study adds to a growing body of research that links responsive caregiving to positive child outcomes and is among the first to use a measure of direct observation in LMIC [25, 37, 38]. Tools that complement parent reporting with live observational strategies may help to capture a more complete picture of responsive caregiving behaviors and of children’s response to those behaviors. We found that responsive caregiving was associated with better child outcomes above and beyond sociodemographic factors, which has been found previously [38]. Furthermore, interventions that focus on responsive stimulation have been shown to improve child outcomes [37, 39–41] indicating that responsive caregiving is a modifiable domain of focus. Therefore, the OMCI may be an ideal tool to guide interventions and studies geared towards optimizing child development through responsive caregiving.

The study found no meaningful association between OMCI and 24-month child height-for-age z-score (HAZ). The current literature provides a mixed picture on this relationship. While some studies have identified a relationship between responsive caregiving and HAZ [4, 42, 43], others have found no effects of responsive stimulation on child growth [39, 40, 44, 45]. In a 2 × 2 factorial randomized controlled trial of responsive stimulation and enhanced nutrition in Pakistan, improvements in child HAZ was only found in the enhanced nutrition arms [44] in which supplements were provided in addition to nutrition education. Other studies in Bangladesh and Uganda have also found no effects of responsive caregiving interventions with emphasis on responsive stimulation and caregiving on linear growth [39, 40, 45]. In our sample, the average HAZ at 24 months was − 1.23 and the prevalence of stunting was around 25%, highlighting significant levels of overall disadvantage for children. In our population, it may be the case that environmental factors, such as latrine use and handwashing, and structural factors, such as food security and wealth, are more important for child HAZ than responsive caregiving alone. In addition, responsive caregiving as captured through mother-child interactions with a picture book in the OMCI may not be an adequate measure of the responsive caregiving behaviors that are pertinent for child nutritional status, such as maternal behavior during feeding.

The strength of the association between the OMCI and the ASQ-SE, as well as the links to sociodemographic variables previously connected to responsive caregiving, demonstrate that the OMCI is predictive of relationships supported by previous research and is worthy of further use in LMIC in assessing responsive caregiving. Based on the comparison with the HOME Inventory, which is often used to assess responsive caregiving in LMIC, the OMCI is capturing unique information. Coded and structured interactions, assessed by the OMCI rather than the unstructured HOME, may be useful in determining specific domains that are linked to development. This information could better serve research and interventions that investigate how specific, and potentially teachable, responsive caregiving behaviors are related to development in ways that have yet to be possible without the addition of this measurement strategy. Further research should compare the OMCI and the HOME, as this study is limited in that the measures were taken at different time points and there could be significant temporal variations between the effects of responsive caregiving at 12 and 24 months. It is likely that using the OMCI and the HOME in tandem could yield a more robust picture of responsive-caregiving and the environment in which it occurs.

## Strengths and limitations

This study has several strengths. First, we used a unique, low-cost tool to assess responsive caregiving that does not rely on maternal self-report or require extensive resources (e.g., video recording equipment, lab space, etc.). Moreover, assessing responsive caregiving in its ‘natural’ environment may be more valid than lab-based settings. Second, we leveraged population-representative longitudinal data to explore the impact of responsive caregiving on child outcomes in an understudied, vulnerable population. Several of the strengths provided by the OMCI also create limitations for this study. Though the OMCI’s direct observation feature is helpful in LMIC where video recording is logistically infeasible or socially inappropriate, it is also limiting in that it is subject to greater rates of observer bias. Though the tool has been shown to demonstrate good inter-observer reliability between expert and trained assessors [4], it is still a limitation that the responsive caregiving interactions are unable to be assessed multiple times in video analysis to ensure consistent coding across multiple assessors. Additionally, the observed responsive caregiving interactions may have been different than their regular responsive caregiving interactions, due to observer effects (though this is also a limitation in other video-based assessments). However, the OMCI tool was designed to reduce observer bias as much as possible [4]. For example, mothers were familiar with the person observing them and the observation occurred in their home. Additionally, we were limited in that nutritional factors such as exclusive breastfeeding status, complementary feeding practices, and dietary diversity, were not available for further analysis. An important aspect of responsive caregiving involves the child feeding behaviors and practices of mothers. Future tools to characterize responsive caregiving should take into account both structured play observation as well as mother-child interactions during feeding. Another limitation is that the responsive caregiving interaction was carried out using a picture book, which may have been a preference for more educated mothers. While the book had no words, it still may have been biased towards mothers who were more familiar with books. Using some other culturally-appropriate toy during the responsive caregiving interaction may be more successful in eliminating bias towards educated mothers. Since the OMCI is a relatively recent tool, it has been used in limited studies since its development in 2013 and warrants further investigations, particularly given the promise it demonstrated with the present analyses.

## Conclusion

This report presents a novel link between OMCI and children’s socioemotional development. Greater responsive-caregiving scores were linked with improved child socioemotional outcomes; and responsive caregiving was found to be associated with lack of maternal depression, higher levels of maternal education, greater assets, and lower numbers of children. The strength of the association between responsive caregiving and improved socioemotional outcomes reinforces the importance of incorporating responsive caregiving in interventions aimed at improving child development. The relationship between higher maternal education and better responsive caregiving indicates, as has been demonstrated and documented extensively [46, 47], that investments in female education will have significant positive effects across generations. Future work can help to further elucidate pathways between responsive caregiving and child outcomes. Though the tool is named for mother-child interactions, future studies using the OMCI can investigate the relationship between responsive caregiving scores of other caregivers (e.g. fathers, siblings, extended family) and development outcomes. The subdomains of the OMCI (Maternal scores and Child scores) could be further explored to assess differences in caregiver and child behaviors in predicting child outcomes.

## Abbreviations

OMCI: Observation of Mother-Child Interaction
LMIC: Low- and -middle-income countries
HOME: Home Observation for Measurement of the Environment
RCT: Randomized controlled trial
PHQ-9: Patient Health Questionnaire-9
HAZ: Height-for-age z-score
ASQ-SE: Ages and Stages Questionnaire, Socioemotional
SES: Socioeconomic status
SCID: Structured Clinical Interview for DSM disorders
CI: Confidence interval
